# Supplementary material for: Canadian Adults with Moderate Intakes of Total Sugars have Greater Intakes of Fibre and Key Micronutrients: Results from the Canadian Community Health Survey 2015 Public Use Microdata File
Source: Nutrients. 2020 Apr 17;12(4):1124. doi: 10.3390/nu12041124 (PMC7230278; doi:10.3390/nu12041124)
Supplement: Supplementary file 1 [file nutrients-12-01124-s001.pdf]

Supplemental Table S1. Food Categories by BNS codes.

Food Categories	BNS Codes	Descriptions
	PASTA, RICE, CEREAL GRAINS AND FLOUR - 01	
1	01A	pasta
1	01B	rice
1	01C	cereal grains and flours
1	130A	those in the ingredient column - we think this is an error in the database, but this is spaghetti (1 food entry)
	WHITE BREADS - 02	
2	02A	white bread
	WHOLEMEAL BREADS - 03	
3	03A	whole wheat bread
3	03B	other whole grains
	OTHER BREADS - 04	
4	04A	rolls, bagels, pita bread, croutons, dumplings, matzo, tortilla
4	04B	crackers and crispbreads
4	04C	muffin and english muffin
4	04D	pancakes and waffles
4	04E	croissants, piecrusts & phyllo dough
4	04F	dry mixes (cakes, muffins, pancakes)
4	219B	those in the ingredient column - we think this is an error in the database, but this is submarine (1 entry)
	WHOLEGRAIN AND HIGH FIBRE BREAKFAST CEREALS - 05	
5	05A	whole grain, oats and high fibre breakfast cereals
	OTHER BREAKFAST CEREALS - 06	
6	06A	breakfast cereals
	COOKIES, BISCUITS AND GRANOLA BARS - 07 (+SUG150B COOKIE)	
7	07A	cookies, commercial
7	07B	biscuits, commercial
7	07C	granola bar
7	150B	cookie
	CAKES, PIES, DANISHES AND OTHER PASTRIES - 08 (+SUG150 RECIPES EXCEPT COOKIE IN ABOVE)	
8	08A	pies, commercial
8	08B	cakes, commercial (frozen cake)
8	08C	danishes, doughnuts and other pastries, commercial
8	150A	cakes, cheesecakes, shortcakes and brownies
8	150C	danishes, turnovers & pastries
8	150D	donuts
8	150E	muffins
8	150F	pies (including pie shell)
8	150G	squares & bars

8	150H	sweet rolls and breads
8	150I	filled crepes, bintzes, cobblers
	FROZEN DAIRY PRODUCTS - 09	
9	09A	ice cream
9	09B	ice milk
9	09C	frozen yoghurt
	MILKS - 10	
10	10A	milk, whole
10	10B	milk, 2%
10	10C	milk, 1%
10	10D	milk, skim
10	10E	milk, evaporated, whole
10	10F	milk, evaporated, 2%
10	10G	milk, evaporated, skim
10	10H	milk, condensed
10	10I	other types of milk (whey, buttermilk)
10	10J	plant-based beverage (soy, almond, coconut)
10	10K	goat and sheep milk
	CREAMS - 13	
13	13A	whipping cream
13	13B	table cream
13	13C	half & half cream
13	13D	sour cream
	CHEESES - 14	
14	14A	cottage cheese
14	14B	cheese, less than 10% B.F.
14	14C	cheese, 10% B.F. to 25% B.F.
14	14D	cheese, more than 25% B.F.
	YOGURTS (NATURAL AND WITH FRUIT) - 15	
15	15A	yoghurts, less than 2% B.F.
15	15B	yoghurts, more than 2.1% B.F.
	EGGS - 16	
16	16A	egg
16	16B	egg substitutes
	BUTTERS - 17	
17	17A	butter
	MARGARINES + OTHER FATS & SPREADS - 18, 20, 21 = 18	
18	18A	regular tub margarine
18	18B	calorie-reduced tub margarine
18	20A	block margarine
18	21A	vegetable oils
18	21B	animal fats
18	21C	shortening
18	227A	those in the ingredient column - we think this is an error in the database, but these are all fats and oils

	RED MEATS - 22, 23, 24, 25, 28, 29, 30, 31, 32 = 22	
**22**	22A	beef, lean only
**22**	22B	beef, lean + fat
**22**	22C	beef, ground
**22**	23A	veal, lean only
**22**	23B	veal, lean + fat (incl ground veal)
**22**	24A	lamb, lean only
**22**	24B	lamb, lean + fat (incl ground lamb)
**22**	25A	pork, fresh, lean only
**22**	25B	pork, fresh, lean + fat
**22**	25C	bacon
**22**	25D	ham, cured, lean only
**22**	25E	ham, cured, lean + fat
**22**	28A	liver
**22**	28B	liver pate
**22**	29A	offal
**22**	31A	game meat
	SAUSAGE + LUNCHEON MEATS (SOME SUGAR ADDED) - 30+32 = 23	
**23**	30A	sausage
**23**	32A	luncheon meat
	POULTRY - 27	
**27**	27A	chicken, meat only
**27**	27B	chicken, meat + skin
**27**	27C	turkey, meat only
**27**	27D	turkey, meat + skin (incl ground turkey)
**27**	27E	other birds (duck, pheasant, pigeon)
**27**	27F	birds, skin only
	NUTS, SEEDS AND PEANUT BUTTER - 33	
**33**	33A	nuts
**33**	33B	seeds
**33**	33C	peanut butter and other nut spreads
	FISH + SHELLFISHES - 34, 35 = 34	
**34**	34A	fish, less than 6% total fat
**34**	34B	fish, superior or equal to 6% total fat
**34**	35A	shellfish
	VEGETABLES - 36	
**36**	36A	beans
**36**	36B	broccoli
**36**	36C	cabbage and kale
**36**	36D	cauliflower
**36**	36E	carrots
**36**	36F	celery
**36**	36G	corn
**36**	36H	lettuces & leafy greans (spinach, mustard greens, etc.)

**36**	36I	mushrooms
**36**	36J	onion, green onions, leeks, garlic
**36**	36K	beans and snow peas
**36**	36L	peppers, red & green
**36**	36M	squashes
**36**	36N	tomatoes
**36**	36O	juices, tomato & vegetable
**36**	36P	other veg (cucumber, immature beans, brussels sprouts, beets, turnips)
	VEG SOUPS - 501	
**501**	50A	soups with vegetables
**501**	50B	soups without vegetables
	LEGUMES - 37	
**37**	37A	legume
**37**	37B	foods made with vegetable proteins (tofu)
	POTATOES, FRIED - 38	
**38**	38A	potato chips
**38**	38B	fried or roasted potatoes
	POTATOES, RAW AND COOKED (EXCLUDING FRIED) - 39	
**39**	39A	potato
	FRUIT - 40	
**40**	40A	citrus fruits
**40**	40B	apple
**40**	40C	banana
**40**	40D	cherries
**40**	40E	grapes and raisins
**40**	40F	melons
**40**	40G	peaches, nectarines
**40**	40H	pears
**40**	40I	pineapple
**40**	40J	plums and prunes
**40**	40K	strawberries
**40**	40L	other fruits (blueberries, dates, kiwis, fruit salads, etc)
	SUGARS, SYRUPS AND PRESERVES - 41	
**41**	41A	sugars (white and brown)
**41**	41B	jams, jellies and marmalade
**41**	41C	other sugars (syrups, molasses, honey, etc)
**41**	41D	sugar substitutes
	SAVORY SNACKS - 42	
**42**	42A	popcorn, plain & pretzels
**42**	42B	salty and high fat snacks (incl tortilla chips)
	CONFECTIONARY - 43 + 44 = 43	
**43**	43A	candies, gums, etc
**43**	43B	ice pop, sherbet
**43**	43C	gelatin, dessert toppings and pudding mixes, commercial

**43**	44A	chocolate bar
	FRUIT JUICE - 45	
**45**	45A	fruit juice
	ALCOHOLIC BEVERAGES - 47, 48, 49 = 47	
**47**	47A	spirits
**47**	47B	liqueurs
**47**	48A	wine
**47**	49A	beer
**47**	49B	ciders and coolers
	CONDIMENTS + GRAVIES – 50+53 =50	
**50**	50C	gravies
**50**	50D	sauces (white, bearnaise, soya, tartar, ketchup, etc)
**50**	50E	salad dressings (with or without oil)
**50**	50F	seasonings (salt, vinegar, etc)
**50**	53A	spices
**50**	53B	others (baking soda, baking power, yeast, etc)
	TEA + COFFEE – 51+231B+231C = 51	
**51**	51A	tea (incl iced tea)
**51**	51B	coffee
**51**	51C	water
**51**	231B	tea (recipe sub-group)
**51**	231C	coffee (recipe sub-group)
	BABYFOOD PRODUCTS - 52	
**52**	52A	babyfood product
**52**	52B	infant formula
	SUPPLEMENTED BARS, SHAKES AND MEAL REPLACEMENTS - 54	
**54**	54A	energy bar
**54**	54B	protein bar and shake
**54**	54C	meal replacements
	SOFT DRINKS - REGULAR – 46A = 60	
**60**	46A	soft drinks - regular
	SOFT DRINKS - DIET -46B = 61	
**61**	46B	soft drinks - diet
	FRUIT DRINKS – 46C = 62	
**62**	46C	fruit drinks
	OTHER BEVERAGES - 46D-G + 231D = 63	
**63**	46D	other beverages (malted milk, chocolate beverage)
**63**	46E	energy drink
**63**	46F	vitamin water
**63**	46G	sports drink
**63**	231D	milk-based beverage (milk shakes, malted milk, hot cocoa, instant breakfast, etc)

Supplemental Table S2. Demographic information of the study population (n=11,817)

	Frequency		Total Sugars (%E)		P-value[b]
	n	%	Mean[a]	SE	
**Sex**					**<0.0001**
Male	5670	48%	18.0	0.2	
Female	6147	52%	19.7	0.2	
**Age/sex groupings**					**0.012**
Male 19 to 30 years	765	6%	17.4	1.1	
Female 19 to 30 years	757	6%	20.7	0.6	
Male 31 to 50 years	1839	16%	18.3	0.4	
Female 31 to 50 years	1945	16%	19.1	0.4	
Male 51 to 70 years	1961	17%	17.5	0.4	
Female 51 to 70 years	2105	18%	19.4	0.3	
Male 71 or older	1105	9%	19.6	0.7	
Female 71 or older	1340	11%	20.8	0.8	
**Type of smoker**					0.759
Daily	1749	15%	19.2	0.5	
Occasionally	540	5%	17.6	0.9	
Not at all	9519	81%	18.8	0.2	
**Self-perceived health**					**0.034**
Excellent	2209	19%	18.4	0.3	
Very good	4391	37%	18.8	0.3	
Good	3705	31%	18.8	0.3	
Fair	1178	10%	20.0	1.0	
Poor	321	3%	20.6	1.9	
**Has high blood pressure**					0.160
Yes	2888	24%	18.4	0.3	
No	8899	75%	18.9	0.2	
**Has diabetes**					**0.006**
Yes	1092	9%	17.3	0.5	
No	10707	91%	18.9	0.2	
**Has heart disease**					0.687
Yes	788	7%	20.1	0.6	
No	10996	93%	18.8	0.2	

**Has cancer**					0.662
Yes	290	2%	19.7	0.8	
No	11504	97%	18.8	0.2	
**Has osteoporosis**[c]					0.507
Yes	793	7%	19.7	1.9	
No	5915	50%	18.9	0.2	
**Body Mass Index**					0.584*
18.5 to <25 kg/m2	3826	32%	18.8	0.4	
25 to <30 kg/m2	4359	37%	19.0	0.3	
30 to <35 kg/m2	2315	20%	19.1	0.4	
35 to <40 kg/m2	850	7%	18.1	1.3	
40 and over kg/m2	467	4%	17.8	0.7	
**Highest level of education**					0.711
Less than high school diploma or its equivalent	1934	16%	19.1	0.9	
High school diploma or a high school equivalency certificate	3043	26%	19.2	0.3	
Certificate/diploma - trade/college/non-university/university below Bachelor's	3924	33%	18.3	0.2	
Bachelor's degree or university certificate/diploma/degree above Bachelor's level	2846	24%	19.0	0.3	
**Income**					0.188
$0 - $19,999	1311	11%	20.1	0.7	
$20,000 - $39,999	2503	21%	18.9	1.1	
$40,000 - $59,999	2114	18%	19.4	0.5	
$60,000 - $79,999	1651	14%	19.3	0.5	
$80,000 - $99,999	1183	10%	18.6	0.5	
$100,000 - $119,999	970	8%	17.7	0.9	
$120,000 - $139,999	617	5%	18.1	0.7	
$140,000 and higher	1461	12%	18.4	0.5	

a adjusted for misreporting
b P-values of the association between covariates and total sugars intake as % of energy, significant if $p<0.05$
c Respondents aged 50 and over
*When assessed as a continuous variable, p= 0.586 for the association with total sugars (%E)
The number of responses which did not indicate a selected option (i.e. "valid skip", "refusal", "don't know", "not stated" responses) were the following: type of smoker (n=9, 0.1%), self-perceived health (n=13, 0.1%), has high blood pressure (n=30, 0.3%), has diabetes (n=18, 0.2%),

has heart disease (n=33, 0.3%), has cancer (n=23, 0.2%), has osteoporosis (n=5109, 43.2%, the majority of which is because this only included responses from women ≥50y), highest level of education (n=70, 0.6%), income (n=7, 0.1%).

Supplementary Table S3. Adjusted means (g/day) and confidence intervals of non-essential food categories and confidence intervals by quintiles of total sugars intake (% energy) in all adults (n=11,817) in Models 1 and 2[*]

	**Q1**				**Q3**				**Q5**				**Model 1**	**Model 2**
	**n=2,364**				**n=2,363**				**n=2,364**					
	**Model 1**		**Model 2**		**Model 1**		**Model 2**		**Model 1**		**Model 2**			
**Food Category**	**Mean**	**95% CI**	**Mean**	**95% CI**	**Mean**	**95% CI**	**Mean**	**95% CI**	**Mean**	**95% CI**	**Mean**	**95% CI**	**p-values[**]**	**p-values[**]**
Creams	8.3	6.0, 10.5	8.4	6.3, 10.4	7.2	5.9, 8.4	7.4	6.1, 8.7	6.8	5.4, 8.1	7.0	5.7, 8.4	0.5611	0.592
Cheeses	36.6	31.6, 41.3	35.8[¥]	31.6, 40.0	25.4	21.9, 28.9	24.7	22.5, 27.0	16.8	13.6, 20.1	17.8	14.7, 21.0	<.0001	0.002
Eggs	33.8	25.5, 42.1	31.7[¥]	25.6, 37.9	24.4	21.4, 27.4	22.9[¥]	19.9, 25.9	15.0	12.8, 17.2	15.3	12.8, 17.9	<.0001	<.0001
Butters	3.3	2.2, 4.4	3.5	2.2, 4.8	3.2	2.6, 3.7	3.3	2.8, 3.8	2.5	1.8, 3.2	2.8	2.0, 3.5	0.0267	0.069
Margarines, other fats, spreads	15.4	12.8, 18.0	14.9[¥]	12.7, 17.1	12.0	11.1, 12.9	11.7[¥]	10.8, 12.5	7.9	7.1, 8.6	8.2	7.5, 8.9	<.0001	<.0001
Red meats	60.6	52.1, 69.0	57.6[¥]	50.2, 65.0	48.9	42.8, 54.9	48.5[¥]	43.3, 53.6	33.3	28.3, 38.2	35.8	31.6, 40.0	<.0001	<.0001
Sausage, luncheon meats	22.2	18.1, 26.4	21.4[¥]	16.6, 26.2	18.4	14.5, 22.4	19.1	16.1, 22.0	13.2	10.5, 16.0	14.5	11.4, 17.6	0.0009	0.006
Poultry	58.0	45.8, 70.2	53.5[¥]	43.3, 63.6	48.2	39.1, 57.2	44.6[¥]	36.5, 52.7	31.4	25.4, 37.4	31.5	24.0, 39.0	<.0001	<.0001
Nuts, seeds, peanut butter	13.4	9.8, 17.1	13.1[¥]	9.9, 16.2	11.4	9.4, 13.4	10.9[¥]	9.3, 12.5	7.1	5.3, 8.8	7.6	5.9, 9.3	<.0001	<.0001
Fish, shellfish	23.3	16.0, 30.7	22.3	16.8, 27.8	20.4	14.4, 26.4	19.2	14.2, 24.2	12.4	7.7, 17.2	12.8	8.6, 17.0	0.0035	0.009
Vegetables, vegetable soups	160.1	145.5, 174.6	157.0	143.8, 170.3	170.3	155.1, 185.4	164.9	148.5, 181.4	168.6	148.6, 188.6	169.5	150.6, 188.5	0.6069	0.629
Legumes	15.5	10.7, 20.2	13.4	9.0, 17.7	13.9	10.8, 16.9	11.6	8.5, 14.6	9.0	4.7, 13.2	8.2	3.7, 12.7	0.161	0.431
Potatoes, fried	19.9	15.5, 24.3	18.8[¥]	14.8, 22.7	15.3	8.4, 22.2	15.2	9.8, 20.5	9.4	7.2, 11.6	9.8	7.6, 12.7	0.0002	0.001
Potatoes, raw and cooked	38.7	32.2, 45.2	41.7[¥]	34.6, 48.8	35.3	30.0, 40.7	38.3	32.6, 44.0	26.2	21.7, 30.6	29.8	24.6, 34.9	<.0001	0.001
Savory snacks	6.5	4.5, 8.5	6.3	4.0, 8.7	4.8	3.5, 6.1	4.5	3.3, 5.7	3.9	2.6, 5.2	4.0	2.8, 5.3	0.4455	0.656
Condiments, gravies	29.5	26.6, 32.3	28.1[¥]	25.2, 31.0	26.9	23.7, 30.0	26.4[¥]	23.9, 28.8	21.6	19.3, 24.0	21.8	19.5, 24.0	0.001	0.003
Supplemented bars, shakes, meal replacements	3.4	2.0, 4.8	3.5	2.1, 4.8	4.5	3.1, 6.0	4.4	2.8, 6.0	5.9	2.9, 8.8	6.0	3.1, 9.0	0.057	0.060
Soups	39.3	32.3, 46.3	41.5	34.2, 48.7	39.6	26.5, 52.7	41.3	27.7, 54.9	41.7	30.8, 52.6	43.1	33.4, 52.8	0.0571	0.088

* Intake of food categories across quintiles of total sugars intake (as % energy) were compared using a general linear model (PROC SURVEYREG) including the following covariates: Model 1, misreporting status (determined using previously published methods by Garriguet [47]); Model 2, misreporting status plus age, sex, smoking, self, perceived health, blood pressure, diabetes, heart disease, cancer, osteoporosis, education, physical activity, income, BMI, immigrant status, weekend reference day and total energy intake. Note: Quintiles 2 and 4 are not presented for readability.

** The p-value represents the global p-value for a significant difference across quintiles of intake of total sugars (%E)

[¥] significant difference compared to Q5 in post-hoc comparison with Bonferroni adjustment in Model 3 ($p<0.05$)

[£] significant difference compared to Q3 in post-hoc comparison with Bonferroni adjustment in Model 3 ($p<0.05$)

CI, confidence interval.

Supplementary Table S4. Means and standard errors of the intakes from all food categories by quintiles of total sugars intake (% energy) in all adults (g/day) (n=11,817)*.

	**Q1**		**Q3**		**Q5**	
	**n=2364**		**n=2363**		**n=2364**	
**Food Category**	**Mean**	**SE**	**Mean**	**SE**	**Mean**	**SE**
Creams	**8.3**	1.2	**7.2**	0.6	**6.8**	0.7
Pasta, rice, cereal grains and flour	**112.3**	14.6	**83.9**	6.2	**53.6**	6.2
White breads	**21.0**	1.7	**31.2**	4.4	**22.9**	1.7
Wholemeal breads	**21.6**	1.8	**21.7**	3.0	**16.6**	1.9
Other breads	**43.9**	3.7	**35.4**	4.9	**26.7**	2.6
Wholegrain and high fibre breakfast cereals	**15.0**	3.6	**19.7**	3.1	**18.6**	1.7
Other breakfast cereals	**1.1**	0.5	**1.9**	0.6	**2.8**	0.6
Cookies, biscuits and granola bars	**7.0**	1.2	**10.1**	1.0	**11.2**	1.0
Cakes, pies, danishes and other pastries	**13.3**	1.8	**30.4**	2.7	**35.7**	3.6
Frozen dairy products	**2.3**	0.6	**16.1**	4.1	**22.6**	2.5
Milks	**58.7**	4.7	**170.7**	8.4	**190.5**	14.8
Yogurts (natural and with fruits)	**11.4**	2.2	**26.8**	4.0	**33.3**	3.2
Fruit	**59.7**	4.0	**160.4**	7.8	**223.6**	10.8
Sugars, syrups, preserves	**4.4**	0.4	**10.7**	0.8	**20.4**	1.9
Confectionary	**2.5**	0.4	**9.1**	1.6	**24.2**	3.4
Fruit juice	**23.4**	3.9	**58.9**	4.0	**112.6**	8.1
Alcoholic beverages	**326.8**	42.1	**116.2**	18.7	**52.5**	9.0
Tea, coffee	**1511.1**	35.7	**1555.6**	48.2	**1308.7**	34.7
Soft drinks, regular	**24.5**	4.5	**60.6**	6.1	**190.4**	26.0
Soft drinks, diet	**55.4**	8.7	**40.6**	11.4	**21.8**	5.4
Fruit drinks	**11.1**[¥]	3.6	**18.2**	4.1	**53.5**	6.9

Other beverages	**3.4**	1.3	**9.5**	4.9	**27.1**	15.5
Cheeses	**36.6**	2.6	**25.4**	1.8	**16.8**	1.7
Eggs	**33.8**	4.2	**24.4**	1.5	**15.0**	1.1
Butters	**3.3**	0.6	**3.2**	0.3	**2.5**	0.4
Margarines, other fats, spreads	**15.4**	1.3	**12.0**	0.5	**7.9**	0.4
Red meats	**60.6**	4.3	**48.9**	3.1	**33.3**	2.5
Sausage, luncheon meats	**22.2**	2.1	**18.4**	2.0	**13.2**	1.4
Poultry	**58.0**	6.2	**48.2**	4.6	**31.4**	3.1
Nuts, seeds, peanut butter	**13.4**	1.9	**11.4**	1.0	**7.1**	0.9
Fish, shellfish	**23.3**	3.7	**20.4**	3.1	**12.4**	2.4
Vegetables, vegetable soups	**160.1**	7.4	**170.3**	7.7	**168.6**	10.2
Legumes	**15.5**	2.4	**13.9**	1.6	**9.0**	2.2
Potatoes, fried	**19.9**	2.2	**15.3**	3.5	**9.4**	1.1
Potatoes, raw and cooked	**38.7**	3.3	**35.3**	2.7	**26.2**	2.3
Savory snacks	**6.5**	1.0	**4.8**	0.6	**3.9**	0.7
Condiments, gravies	**29.5**	1.5	**26.9**	1.6	**21.6**	1.2
Baby food products	**0.1**	0.2	**0.3**	0.3	**0.2**	0.8
Supplemented bars, shakes, meal replacements	**3.4**	0.7	**4.5**	0.7	**5.9**	1.5
Soups	**39.3**	3.6	**39.6**	6.7	**41.7**	5.5

SE, standard error.

Table S5. Means and standard errors of intakes from macro- and micronutrients by quintiles of total sugars intake (% energy) in all adults (n=11,817)*.

	Q1		Q3		Q5		ALL	
	n=2364		n=2363		n=2364		n=11,817	
	**Mean**	**SE**	**Mean**	**SE**	**Mean**	**SE**	**Mean**	**SE**
Energy (kcal)	**1953.8**	25.4	**1919.2**[¥]	50.5	**1805.5**	21.2	**1890.1**	20.4
Carbohydrates (%)	**38.9**[¥£]	0.8	**47.7**[¥]	0.9	**58.4**	0.4	**47.7**	0.7
Total Fibre (g/1000 kcal)	**8.6**[¥£]	0.2	**10.1**	0.4	**10.3**	0.2	**9.7**	0.2
Total Sugars (%)	**7.9**[¥£]	0.1	**18.3**[¥]	0.01	**33.0**	0.3	**18.8**	0.2
Natural Sugars (%)	**4.8**[¥£]	0.1	**10.2**[¥]	0.2	**15.6**	0.3	**10.1**	0.1
Added Sugars (%)	**3.0**[¥£]	0.1	**8.1**[¥]	0.3	**17.3**	0.4	**8.6**	0.1
Free Sugars (%)	**3.2**[¥£]	0.1	**9.2**[¥]	0.3	**20.1**	0.4	**9.9**	0.1
Fat (%)	**36.1**[¥£]	0.5	**32.2**[¥]	0.5	**26.4**	0.4	**32.2**	0.6
Saturated Fat (%)	**11.3**[¥£]	0.2	**10.4**[¥]	0.3	**9.0**	0.1	**10.4**	0.2
Monounsaturated Fat (%)	**13.8**[¥£]	0.2	**12.0**[¥]	0.3	**9.4**	0.2	**12.0**	0.2
Polyunsaturated Fat (%)	**7.8**[¥£]	0.2	**6.8**[¥]	0.1	**5.5**	0.1	**6.9**	0.2
Linoleic acid (%)	**6.6**[¥£]	0.2	**5.7**[¥]	0.1	**4.6**	0.1	**5.9**	0.2
Alpha-linolenic Acid (%)	**0.8**[¥]	0.01	**0.8**[¥]	0.01	**0.6**	0.01	**0.7**	0.01
Protein (%)	**18.8**[¥]	0.4	**17.5**[¥]	0.2	**13.9**	0.3	**17.0**	0.2
Cholesterol (mg/1000 kcal)	**178.1**[¥]	7.9	**153.2**[¥]	7.0	**109.9**	4.4	**147.0**	2.1
Alcohol (%)	**6.2**[¥£]	0.7	**2.6**[¥]	0.3	**1.3**	0.3	**3.1**	0.3
Vitamin A (ug RAE)	**338.9**[£]	20.6	**370.6**	10.6	**378.7**	19.3	**367.9**	10.3
Thiamin (mg)	**0.9**	0.01	**0.9**	0.01	**0.8**	0.01	**0.9**	0.0
Riboflavin (mg)	**1.0**[£]	0.01	**1.1**	0.01	**1.1**	0.01	**1.1**	0.0
Niacin (mg)	**23.7**[¥£]	0.6	**21.7**[¥]	0.4	**18.0**	0.3	**21.4**	0.3
Vitamin B-6 (mg)	**0.9**	0.01	**1.0**	0.0	**0.9**	0.01	**0.9**	0.01
Folate (ug DFE)	**247.8**[¥]	4.6	**242.8**[¥]	7.5	**221.9**	5.7	**240.7**	2.5
Vitamin B-12 (mg)	**2.3**	0.1	**2.4**[¥]	0.1	**1.9**	0.1	**2.2**	0.1

Vitamin C (mg)	**35.9**¥£	1.8	**56.2**	3.0	**85.1**	5.7	**56.4**	1.5
Vitamin D (ug)	**2.3**¥£	0.2	**2.7**	0.1	**2.6**	0.1	**2.6**	0.1
Calcium (mg)	**370.8**¥£	11.7	**439.8**	9.2	**458.2**	8.5	**428.1**	5.6
Iron (mg)	**6.7**¥	0.1	**6.9**¥	0.1	**6.3**	0.1	**6.8**	0.01
Magnesium (mg)	**167.3**	3.1	**177.7**	3.5	**169.7**	2.9	**172.9**	1.2
Phosphorus (mg)	**696.2**¥	12.4	**704.8**¥	8.2	**652.0**	8.4	**696.7**	4.7
Potassium (mg)	**1378.1**¥£	21.4	**1536.7**	20.9	**1611.8**	26.2	**1511.2**	10.1
Zinc (mg)	**6.0**¥	0.1	**5.7**¥	0.1	**4.8**	0.1	**5.6**	0.1
Sodium (mg)	**1621.9**¥£	27.5	**1512.0**¥	19.6	**1255.1**	20.2	**1478.9**	11.0

* Intake of macro- and micronutrients across quintiles of total sugars intake (as % energy) were compared using a general linear model (PROC SURVEYREG) including the following covariates: Model 1-misreporting status (determined using previously published methods by Garriguet [47]); Model 3- misreporting status plus age, sex, smoking, self-perceived health, blood pressure, diabetes, heart disease, cancer, osteoporosis, education, physical activity, income, BMI, immigrant status and weekend reference day. Note: Quintiles 2 and 4 are not presented for readability.

RAE, retinoic acid equivalent; DFE, dietary folate equivalents; SE, standard error.

Supplemental Table S6. Comparison of intakes from macro- and micronutrients by quintiles of total sugars intake (% energy) in Males Aged 19-30y*

**Males 19-30y (n=765)**	**Q1**		**Q3**		**Q5**		
	**Mean**	**SE**	**Mean**	**SE**	**Mean**	**SE**	**p-value****
Energy (kcal)	**2379.9**	106.5	**2608.3**	135.9	**2338.3**	121.7	0.028
Carbohydrates (%)	**38.8**[¥£]	1.9	**45.1**[¥]	1.8	**58.2**	1.1	<.0001
Total Fibre (g/1000 kcal)	**6.8**	0.4	**7.5**	0.7	**7.7**	0.9	0.108
Total Sugars (%)	**7.9**[¥£]	0.3	**18.4**[¥]	0.2	**32.3**	1.1	<.0001
Natural Sugars (%)	**4.3**[¥£]	0.4	**8.8**	0.8	**12.1**	2.1	<.0001
Added Sugars (%)	**3.5**[¥£]	0.3	**9.6**[¥]	0.9	**20.2**	2.2	<.0001
Free Sugars (%)	**3.8**[¥£]	0.3	**11.2**[¥]	0.7	**23.0**	2.1	<.0001
Fat (%)	**35.2**[¥]	2.2	**33.5**[¥]	1.1	**26.4**	0.9	<.0001
Saturated Fat (%)	**11.2**	1.0	**11.2**	1.1	**10.0**	0.6	0.029
Monounsaturated Fat (%)	**13.4**[¥]	0.8	**12.4**[¥]	0.4	**9.0**	0.5	<.0001
Polyunsaturated Fat (%)	**7.5**[¥]	0.3	**6.5**[¥]	0.9	**4.8**	0.3	<.0001
Linoleic acid (%)	**6.5**[¥]	0.2	**5.7**[¥]	0.6	**4.1**	0.2	<.0001
Alpha-linonelic Acid (%)	**0.8**[¥]	0.1	**0.7**	0.1	**0.6**	0.0	0.003
Protein (%)	**19.4**[¥]	1.8	**17.9**[¥]	0.7	**14.5**	0.6	<.0001
Cholesterol (mg/1000 kcal)	**192.8**	41.9	**166.0**	15.6	**107.6**	11.6	0.038
Alcohol (%)	**6.6**[¥]	2.1	**3.6**	1.1	**0.9**	0.6	0.008
**micronutrients: per 1000 kcal**							
Vitamin A (ug RAE)	**323.8**	36.9	**313.3**	49.7	**289.5**	40.7	0.867
Thiamin (mg)	**0.9**	0.1	**0.7**	0.1	**0.9**	0.0	0.375
Riboflavin (mg)	**1.0**	0.0	**1.1**	0.0	**1.1**	0.1	0.399
Niacin (mg)	**23.6**	2.0	**22.6**	1.1	**20.7**	1.1	0.513
Vitamin B-6 (mg)	**0.9**	0.0	**1.0**	0.1	**1.1**	0.1	0.509

Folate (ug DFE)	**269.3**	19.9	**230.5**	21.9	**224.2**	16.4	0.281
Vitamin B-12 (mg)	**2.0**	0.3	**2.3**	0.2	**2.0**	0.1	0.435
Vitamin C (mg)	**29.1**¥£	4.2	**48.8**	10.6	**72.8**	10.1	<.0001
Vitamin D (ug)	**1.7**	0.3	**2.1**	0.5	**2.6**	0.4	0.372
Calcium (mg)	**404.4**	25.6	**437.3**	26.2	**433.7**	43.8	0.976
Iron (mg)	**6.3**	0.3	**6.3**	0.6	**6.3**	0.4	0.977
Magnesium (mg)	**145.1**	6.1	**152.1**	11.2	**142.7**	13.0	0.558
Phosphorus (mg)	**686.1**	18.8	**680.9**	26.0	**624.8**	36.2	0.487
Potassium (mg)	**1209.2**¥	55.1	**1293.2**	83.1	**1368.3**	77.9	0.004
Zinc (mg)	**5.5**	0.5	**5.7**	0.5	**4.7**	0.4	0.026
Sodium (mg)	**1797.3**¥	144.9	**1485.3**¥	76.9	**1280.0**	68.8	0.001

* Intake of macro- and micronutrients across quintiles of total sugars intake (as % energy) were compared using a general linear model (PROC SURVEYREG) including the following covariates: Model 3, mis-reporting status (determined using previously published methods by Garriguet [47]), age, smoking, self-perceived health, blood pressure, diabetes, heart disease, cancer, osteoporosis, education, physical activity, income, BMI, immigrant status and weekend reference day. Note: Quintiles 2 and 4 are not presented for readability.

** The p-value represents the global p-value for a significant difference across quintiles of intake of total sugars (%E)

¥ significant difference compared to Q5 in post-hoc comparison with Bonferroni adjustment based in Model 3 (p<0.05)

£ significant difference compared to Q3 in post-hoc comparison with Bonferroni adjustment in Model 3 (p<0.05)

BMI, body mass index; DFE, dietary folate equivalents; y, years

Supplemental Table S7. Comparison of intakes from macro- and micronutrients by quintiles of total sugars intake (% energy) in Females Aged 19-30y*

**Females 19-30y (n=757)**	**Q1**		**Q3**		**Q5**		
	**Mean**	**SE**	**Mean**	**SE**	**Mean**	**SE**	**p-value****
Energy (kcal)	**1650.9**	130.5	**1694.9**	67.0	**1676.8**	56.4	0.630
Carbohydrates (%)	**37.6¥£**	2.2	**47.3¥**	1.4	**60.0**	1.6	<.0001
Total Fibre (g/1000 kcal)	**8.5**	1.4	**9.9**	0.7	**9.3**	0.7	0.461
Total Sugars (%)	**7.6¥£**	0.6	**18.5¥**	0.2	**33.8**	1.1	<.0001
Natural Sugars (%)	**5.6¥**	1.5	**9.9¥**	1.1	**16.4**	1.6	<.0001
Added Sugars (%)	**2.0¥£**	1.0	**8.6¥**	1.1	**17.3**	1.3	<.0001
Free Sugars (%)	**2.1¥£**	1.1	**10.0¥**	0.9	**21.1**	1.7	<.0001
Fat (%)	**34.6¥**	2.3	**33.0¥**	1.4	**25.6**	1.4	<.0001
Saturated Fat (%)	**11.2**	1.3	**10.5**	0.5	**8.9**	0.6	0.004
Monounsaturated Fat (%)	**13.3¥**	0.9	**12.4¥**	0.7	**9.1**	0.4	<.0001
Polyunsaturated Fat (%)	**6.9**	0.5	**7.0¥**	0.7	**5.2**	0.5	<.0001
Linoleic acid (%)	**5.9**	0.4	**5.9¥**	0.4	**4.4**	0.4	<.0001
Alpha-linonelic Acid (%)	**0.7**	0.1	**0.7**	0.1	**0.6**	0.1	0.059
Protein (%)	**18.9¥**	1.9	**18.4¥**	1.0	**13.7**	0.6	<.0001
Cholesterol (mg/1000 kcal)	**164.3¥**	15.5	**166.0¥**	15.8	**96.9**	9.6	<.0001
Alcohol (%)	**8.9**	3.7	**1.3**	0.5	**0.6**	0.5	0.244
**micronutrients: per 1000 kcal**							
Vitamin A (ug RAE)	**449.3**	170.5	**316.3**	64.4	**412.0**	51.6	0.571
Thiamin (mg)	**0.9**	0.1	**0.9**	0.1	**0.8**	0.0	0.337
Riboflavin (mg)	**1.1**	0.1	**1.1**	0.0	**1.1**	0.1	0.562
Niacin (mg)	**23.8¥**	2.3	**23.7¥**	1.3	**17.3**	1.2	<.0001
Vitamin B-6 (mg)	**0.9**	0.1	**1.0**	0.1	**1.0**	0.1	0.140

Folate (ug DFE)	**287.7**	44.1	**255.5**	13.8	**219.1**	13.6	0.090
Vitamin B-12 (mg)	**2.8**	0.4	**1.9**	0.3	**2.1**	0.3	0.739
Vitamin C (mg)	**43.7**	16.4	**58.0**	9.2	**81.7**	8.9	0.011
Vitamin D (ug)	**2.3**	0.8	**2.7**	0.9	**2.5**	0.4	0.408
Calcium (mg)	**420.2**	62.9	**475.7**	32.4	**496.7**	24.8	0.022
Iron (mg)	**6.7**	0.4	**6.3**	0.5	**6.3**	0.3	0.444
Magnesium (mg)	**167.3**	22.8	**163.7**	7.0	**168.5**	8.1	0.656
Phosphorus (mg)	**678.0**	23.2	**703.6**	27.9	**659.0**	29.3	0.831
Potassium (mg)	**1420.4**	135.2	**1495.1**	71.1	**1593.9**	75.8	0.122
Zinc (mg)	**6.5**	0.8	**4.7**	0.3	**4.9**	0.3	<.0001
Sodium (mg)	**1585.4**	120.2	**1393.2**	107.8	**1258.7**	65.2	0.029

* Intake of macro- and micronutrients across quintiles of total sugars intake (as % energy) were compared using a general linear model (PROC SURVEYREG) including the following covariates: Model 3, mis-reporting status (determined using previously published methods by Garriguet [47]), age, smoking, self-perceived health, blood pressure, diabetes, heart disease, cancer, osteoporosis, education, physical activity, income, BMI, immigrant status and weekend reference day. Note: Quintiles 2 and 4 are not presented for readability.

** The p-value represents the global p-value for a significant difference across quintiles of intake of total sugars (%E)

¥ significant difference compared to Q5 in post-hoc comparison with Bonferroni adjustment in Model 3 ($p<0.05$)

£ significant difference compared to Q3 in post-hoc comparison with Bonferroni adjustment in Model 3 ($p<0.05$)

BMI, body mass index; DFE, dietary folate equivalents; y, years

Supplemental Table S8. Comparison of intakes from macro- and micronutrients by quintiles of total sugars intake (% energy) in Males Aged 31-50y*

**Males 31-50y (n=1839)**	**Q1**		**Q3**		**Q5**		
	**Mean**	**SE**	**Mean**	**SE**	**Mean**	**SE**	**p-value****
Energy (kcal)	**2316.8**	56.3	**2313.9**	67.4	**2131.7**	128.2	0.028
Carbohydrates (%)	**38.4**[¥£]	1.2	**48.3**[¥]	0.9	**58.7**	0.9	<.0001
Total Fibre (g/1000 kcal)	**8.2**	0.5	**9.2**	0.5	**9.0**	0.7	0.043
Total Sugars (%)	**7.7**[¥£]	0.3	**18.3**[¥]	0.1	**34.6**	0.9	<.0001
Natural Sugars (%)	**4.6**[¥£]	0.2	**9.7**	0.7	**13.3**	1.1	<.0001
Added Sugars (%)	**3.0**[¥£]	0.2	**8.6**[¥]	0.7	**21.0**	1.3	<.0001
Free Sugars (%)	**3.3**[¥£]	0.3	**9.7**[¥]	0.7	**23.4**	1.2	<.0001
Fat (%)	**35.9**[¥]	1.0	**32.1**[¥]	1.0	**26.6**	0.8	<.0001
Saturated Fat (%)	**10.9**[¥]	0.3	**10.7**	0.6	**8.7**	0.8	0.020
Monounsaturated Fat (%)	**14.2**[¥£]	0.6	**12.1**[¥]	0.5	**9.9**	0.4	<.0001
Polyunsaturated Fat (%)	**7.6**[¥]	0.3	**6.4**	0.2	**5.5**	0.3	<.0001
Linoleic acid (%)	**6.5**[¥]	0.3	**5.4**	0.2	**4.8**	0.2	<.0001
Alpha-linonelic Acid (%)	**0.7**[¥]	0.0	**0.7**[¥]	0.0	**0.6**	0.0	0.006
Protein (%)	**19.2**[¥]	0.6	**17.3**[¥]	0.5	**13.1**	0.6	<.0001
Cholesterol (mg/1000 kcal)	**180.4**[¥]	22.3	**145.1**	7.8	**108.4**	8.3	<.0001
Alcohol (%)	**6.4**[¥£]	0.7	**2.3**	0.5	**1.7**	0.6	<.0001
**micronutrients: per 1000 kcal**							
Vitamin A (ug RAE)	**346.8**	35.3	**300.3**	14.4	**282.6**	23.8	0.268
Thiamin (mg)	**0.9**	0.0	**0.8**	0.0	**0.8**	0.1	0.538
Riboflavin (mg)	**1.0**	0.1	**1.0**	0.0	**1.0**	0.1	0.278
Niacin (mg)	**24.6**[¥]	0.7	**21.3**[¥]	1.0	**16.8**	0.4	<.0001
Vitamin B-6 (mg)	**1.0**[¥]	0.0	**0.9**	0.1	**0.8**	0.0	<0.001
Folate (ug DFE)	**246.9**	12.0	**233.0**[¥]	12.2	**201.5**	10.6	0.006

Vitamin B-12 (mg)	**2.6**	0.4	**2.3**	0.5	**1.7**	0.2	0.524
Vitamin C (mg)	**37.6**	7.6	**48.0**	3.6	**74.4**	13.9	0.001
Vitamin D (ug)	**2.2**	0.8	**2.3**	0.3	**2.2**	0.3	0.189
Calcium (mg)	**369.9**	22.6	**400.9**	20.6	**397.1**	57.3	0.100
Iron (mg)	**6.6**	0.3	**6.7**	0.2	**5.9**	0.3	0.047
Magnesium (mg)	**165.7**	5.2	**166.6**	4.8	**151.3**	6.6	0.010
Phosphorus (mg)	**686.5**	21.5	**675.1**	21.4	**597.6**	34.0	0.153
Potassium (mg)	**1358.1**	39.0	**1422.0**	34.9	**1465.7**	67.4	0.293
Zinc (mg)	**6.2**	0.4	**5.6**¥	0.2	**4.6**	0.2	0.022
Sodium (mg)	**1519.5**¥	46.8	**1502.0**¥	55.6	**1241.7**	55.5	0.002

* Intake of macro- and micronutrients across quintiles of total sugars intake (as % energy) were compared using a general linear model (PROC SURVEYREG) including the following covariates: Model 3, mis-reporting status (determined using previously published methods by Garriguet [47]), age, smoking, self-perceived health, blood pressure, diabetes, heart disease, cancer, osteoporosis, education, physical activity, income, BMI, immigrant status and weekend reference day. Note: Quintiles 2 and 4 are not presented for readability.

** The p-value represents the global p-value for a significant difference across quintiles of intake of total sugars (%E)

¥ significant difference compared to Q5 in post-hoc comparison with Bonferroni adjustment in Model 3 ($p<0.05$)

£ significant difference compared to Q3 in post-hoc comparison with Bonferroni adjustment in Model 3($p<0.05$)

BMI, body mass index; DFE, dietary folate equivalents; y, years

Supplemental Table S9. Comparison of intakes from macro- and micronutrients by quintiles of total sugars intake (% energy) in Females Aged 31-50y*

**Females 31-50y (n=1945)**	**Q1**		**Q3**		**Q5**		
	**Mean**	**SE**	**Mean**	**SE**	**Mean**	**SE**	**p-value****
Energy (kcal)	**1673.9**	38.2	**1684.2**	41.6	**1618.8**	27.8	0.592
Carbohydrates (%)	**40.0¥£**	1.8	**48.4¥**	1.6	**57.6**	1.0	<.0001
Total Fibre (g/1000 kcal)	**9.4**	0.5	**10.7**	1.0	**11.1**	0.7	0.247
Total Sugars (%)	**8.1¥£**	0.3	**18.3¥**	0.1	**32.6**	0.6	<.0001
Natural Sugars (%)	**4.8¥£**	0.2	**10.3¥**	0.5	**16.1**	0.7	<.0001
Added Sugars (%)	**3.2¥£**	0.2	**7.8¥**	0.4	**16.5**	0.9	<.0001
Free Sugars (%)	**3.6¥£**	0.4	**8.8¥**	0.4	**19.1**	0.9	<.0001
Fat (%)	**37.4¥£**	1.6	**32.4¥**	1.4	**26.8**	0.9	<.0001
Saturated Fat (%)	**12.1¥**	1.0	**10.4¥**	0.5	**9.1**	0.3	<.0001
Monounsaturated Fat (%)	**14.0¥£**	0.7	**12.0¥**	0.5	**9.5**	0.3	<.0001
Polyunsaturated Fat (%)	**7.8¥**	0.3	**7.1¥**	0.3	**5.7**	0.3	<.0001
Linoleic acid (%)	**6.7¥**	0.3	**5.8¥**	0.2	**4.9**	0.3	<.0001
Alpha-linonelic Acid (%)	**0.8**	0.1	**0.8**	0.0	**0.6**	0.0	0.003
Protein (%)	**18.1**	0.8	**17.3¥**	0.4	**15.0**	0.6	<0.001
Cholesterol (mg/1000 kcal)	**169.5**	15.2	**154.8**	24.4	**120.1**	11.4	0.002
Alcohol (%)	**4.5¥**	0.8	**1.8**	0.4	**0.6**	0.2	0.001
**micronutrients: per 1000 kcal**							
Vitamin A (ug RAE)	**309.3**	24.1	**390.9**	34.1	**431.8**	64.3	0.103
Thiamin (mg)	**0.9**	0.0	**0.9**	0.1	**0.8**	0.0	0.062
Riboflavin (mg)	**1.0£**	0.0	**1.1**	0.0	**1.1**	0.0	0.027
Niacin (mg)	**23.1**	1.3	**20.9**	1.2	**19.4**	1.0	<.0001

Vitamin B-6 (mg)	**0.9**	0.1	**0.9**	0.0	**1.0**	0.1	0.629
Folate (ug DFE)	**255.0**	15.4	**262.7**	14.8	**248.8**	14.6	0.417
Vitamin B-12 (mg)	**1.9**	0.2	**2.8**	0.6	**1.9**	0.2	0.017
Vitamin C (mg)	**38.2**¥	3.8	**59.9**	4.6	**97.3**	20.0	<.0001
Vitamin D (ug)	**2.5**	0.4	**2.8**	0.3	**2.2**	0.2	0.107
Calcium (mg)	**376.2**£	17.0	**499.4**	43.0	**451.7**	20.5	<.0001
Iron (mg)	**6.8**	0.2	**7.4**¥	0.3	**6.3**	0.2	0.005
Magnesium (mg)	**167.7**	6.7	**195.7**	9.9	**177.7**	10.0	0.247
Phosphorus (mg)	**725.5**	29.1	**727.2**	23.8	**651.2**	22.9	0.045
Potassium (mg)	**1319.4**¥£	35.7	**1565.9**	45.0	**1664.7**	101.4	<.0001
Zinc (mg)	**5.6**¥	0.2	**5.8**¥	0.2	**4.8**	0.2	0.001
Sodium (mg)	**1666.0**¥	78.9	**1558.5**¥	70.4	**1276.4**	57.8	<0.001

* Intake of macro- and micronutrients across quintiles of total sugars intake (as % energy) were compared using a general linear model (PROC SURVEYREG) including the following covariates: Model 3, mis-reporting status (determined using previously published methods by Garriguet [47]), age, smoking, self-perceived health, blood pressure, diabetes, heart disease, cancer, osteoporosis, education, physical activity, income, BMI, immigrant status and weekend reference day. Note: Quintiles 2 and 4 are not presented for readability.

** The p-value represents the global p-value for a significant difference across quintiles of intake of total sugars (%E)

¥ significant difference compared to Q5 in post-hoc comparison with Bonferroni adjustment in Model 3 (p<0.05)

£ significant difference compared to Q3 in post-hoc comparison with Bonferroni adjustment in Model 3 (p<0.05)

BMI, body mass index; DFE, dietary folate equivalents; y, years

Supplemental Table S10. Comparison of intakes from macro- and micronutrients by quintiles of total sugars intake (% energy) in Males Aged 51-70y*

**Males 51-70y (n=1961)**	**Q1**		**Q3**		**Q5**		
	**Mean**	**SE**	**Mean**	**SE**	**Mean**	**SE**	**p-value****
Energy (kcal)	**1993.6**	34.8	**2140.7**	62.5	**2063.3**	57.9	0.127
Carbohydrates (%)	**38.4¥£**	1.3	**46.7¥**	1.4	**57.8**	0.8	<.0001
Total Fibre (g/1000 kcal)	**8.2¥**	0.3	**9.3**	1.1	**9.7**	0.5	<.0001
Total Sugars (%)	**7.7¥£**	0.2	**18.5¥**	0.2	**33.0**	0.6	<.0001
Natural Sugars (%)	**4.9¥£**	0.3	**10.0¥**	0.5	**15.1**	0.9	<.0001
Added Sugars (%)	**2.7¥£**	0.2	**8.3¥**	0.6	**17.9**	1.1	<.0001
Free Sugars (%)	**3.1¥£**	0.2	**9.3¥**	0.6	**20.5**	0.8	<.0001
Fat (%)	**35.9¥£**	1.4	**32.7¥**	0.9	**27.6**	1.3	<.0001
Saturated Fat (%)	**10.8**	0.5	**10.6**	0.3	**9.5**	0.6	0.101
Monounsaturated Fat (%)	**13.7¥£**	0.5	**12.1¥**	0.4	**9.8**	0.6	<.0001
Polyunsaturated Fat (%)	**8.2¥**	0.4	**7.1¥**	0.3	**5.7**	0.4	<.0001
Linoleic acid (%)	**7.0¥**	0.4	**6.0¥**	0.3	**4.8**	0.3	<.0001
Alpha-linonelic Acid (%)	**0.9**	0.1	**0.8**	0.1	**0.7**	0.1	0.025
Protein (%)	**18.9¥**	0.7	**18.0¥**	0.8	**13.4**	1.3	<.0001
Cholesterol (mg/1000 kcal)	**188.8¥**	13.1	**174.3**	15.0	**112.2**	24.3	0.003
Alcohol (%)	**6.8¥**	0.9	**2.7¥**	0.3	**1.2**	0.3	<.0001
**micronutrients: per 1000 kcal**							
Vitamin A (ug RAE)	**336.7**	31.5	**350.4**	25.1	**386.5**	38.4	0.032
Thiamin (mg)	**0.8**	0.1	**0.8**	0.0	**0.8**	0.1	0.593
Riboflavin (mg)	**1.0**	0.0	**1.1**	0.0	**1.1**	0.0	0.374
Niacin (mg)	**24.0¥**	0.8	**22.1¥**	0.8	**17.7**	0.7	<.0001

Vitamin B-6 (mg)	**0.9**	0.0	**1.0**	0.0	**0.8**	0.1	0.040
Folate (ug DFE)	**235.0**	13.0	**227.5**	8.0	**215.9**	10.7	0.739
Vitamin B-12 (mg)	**2.5**	0.2	**2.4**	0.2	**1.9**	0.3	0.451
Vitamin C (mg)	**34.0**[¥£]	2.8	**45.1**[¥]	8.2	**83.6**	5.8	<.0001
Vitamin D (ug)	**2.6**	0.3	**3.2**	0.4	**3.0**	0.4	0.142
Calcium (mg)	**338.3**[¥£]	17.7	**405.8**	15.2	**450.3**	26.5	<.0001
Iron (mg)	**6.6**	0.2	**6.7**	0.1	**6.2**	0.3	0.018
Magnesium (mg)	**168.2**	5.8	**166.7**	13.8	**172.6**	8.5	0.171
Phosphorus (mg)	**668.1**	13.9	**704.3**	14.1	**679.8**	28.6	0.071
Potassium (mg)	**1403.9**[¥£]	30.3	**1536.4**	34.2	**1592.7**	66.8	0.004
Zinc (mg)	**6.2**[¥]	0.2	**5.9**[¥]	0.2	**4.6**	0.4	<.0001
Sodium (mg)	**1619.8**[¥]	59.0	**1504.1**	46.9	**1277.7**	68.4	0.010

* Intake of macro- and micronutrients across quintiles of total sugars intake (as % energy) were compared using a general linear model (PROC SURVEYREG) including the following covariates: Model 3, mis-reporting status (determined using previously published methods by Garriguet [47]), age, smoking, self-perceived health, blood pressure, diabetes, heart disease, cancer, osteoporosis, education, physical activity, income, BMI, immigrant status and weekend reference day. Note: Quintiles 2 and 4 are not presented for readability.

** The p-value represents the global p-value for a significant difference across quintiles of intake of total sugars (%E)

¥ significant difference compared to Q5 in post-hoc comparison with Bonferroni adjustment in Model 3 (p<0.05)

£ significant difference compared to Q3 in post-hoc comparison with Bonferroni adjustment in Model 3 (p<0.05)

BMI, body mass index; DFE, dietary folate equivalents; y, years

Supplemental Table S11. Comparison of intakes from macro- and micronutrients by quintiles of total sugars intake (% energy) in Females Aged 51-70y*

**Females 51-70y (n=2105)**	**Q1**		**Q3**		**Q5**		
	**Mean**	**SE**	**Mean**	**SE**	**Mean**	**SE**	**p-value****
Energy (kcal)	**1614.0**	35.3	**1567.6**	31.4	**1626.4**	101.2	0.531
Carbohydrates (%)	**38.7[¥£]**	1.1	**48.5[¥]**	0.7	**57.8**	1.3	<.0001
Total Fibre (g/1000 kcal)	**9.5**	0.4	**12.5**	1.0	**11.1**	0.8	<.0001
Total Sugars (%)	**7.9[¥£]**	0.2	**18.3[¥]**	0.1	**32.3**	0.6	<.0001
Natural Sugars (%)	**5.0[¥£]**	0.3	**11.2[¥]**	0.4	**16.4**	1.1	<.0001
Added Sugars (%)	**2.9[¥£]**	0.2	**7.0[¥]**	0.3	**15.8**	0.9	<.0001
Free Sugars (%)	**3.0[¥£]**	0.2	**7.7[¥]**	0.3	**18.4**	0.9	<.0001
Fat (%)	**36.9[¥£]**	0.9	**30.2[¥]**	1.0	**25.8**	0.8	<.0001
Saturated Fat (%)	**11.6[¥£]**	0.4	**9.5**	0.5	**8.7**	0.3	<.0001
Monounsaturated Fat (%)	**14.0[¥£]**	0.4	**11.4[¥]**	0.3	**9.0**	0.3	<.0001
Polyunsaturated Fat (%)	**8.2[¥]**	0.4	**6.7**	0.3	**5.5**	0.3	<.0001
Linoleic acid (%)	**6.8[¥]**	0.3	**5.6**	0.2	**4.7**	0.3	<.0001
Alpha-linonelic Acid (%)	**1.0[¥]**	0.1	**0.8**	0.1	**0.7**	0.0	<0.001
Protein (%)	**18.6[¥]**	0.6	**17.8[¥]**	0.7	**14.2**	0.3	<.0001
Cholesterol (mg/1000 kcal)	**167.0[¥]**	9.3	**138.6[¥]**	9.1	**103.0**	5.9	<.0001
Alcohol (%)	**5.8[¥]**	0.9	**3.5**	1.0	**2.2**	0.8	0.001
**micronutrients: per 1000 kcal**							
Vitamin A (ug RAE)	**331.4**	22.3	**470.9**	41.5	**381.9**	19.4	0.054
Thiamin (mg)	**0.8**	0.1	**0.9**	0.1	**0.8**	0.0	0.110
Riboflavin (mg)	**1.0**	0.0	**1.1**	0.0	**1.1**	0.1	0.075
Niacin (mg)	**23.3[¥]**	0.9	**22.4[¥]**	0.9	**18.0**	0.5	<.0001

Vitamin B-6 (mg)	**0.9**	0.0	**1.0**	0.1	**0.9**	0.0	0.398
Folate (ug DFE)	**234.6**	15.0	**246.6**	15.9	**211.9**	24.9	0.615
Vitamin B-12 (mg)	**2.2**	0.1	**2.2**	0.2	**1.8**	0.1	0.250
Vitamin C (mg)	**35.1¥£**	2.7	**74.0**	5.1	**82.3**	14.4	<.0001
Vitamin D (ug)	**2.5**	0.3	**2.7**	0.3	**2.7**	0.2	0.148
Calcium (mg)	**370.3**	19.4	**441.1**	23.5	**481.3**	27.5	0.001
Iron (mg)	**7.1**	0.2	**7.4**	0.3	**6.4**	0.3	0.002
Magnesium (mg)	**184.4**	6.2	**200.8**	6.8	**177.3**	16.4	0.323
Phosphorus (mg)	**729.3**	26.8	**730.2**	22.6	**659.9**	32.3	0.168
Potassium (mg)	**1513.8**	77.3	**1745.3**	40.7	**1676.4**	113.8	0.016
Zinc (mg)	**6.2¥**	0.2	**6.1¥**	0.3	**5.0**	0.1	<.0001
Sodium (mg)	**1579.2¥**	75.7	**1511.9¥**	65.3	**1185.2**	59.8	<.0001

* Intake of macro- and micronutrients across quintiles of total sugars intake (as % energy) were compared using a general linear model (PROC SURVEYREG) including the following covariates: Model 3, mis-reporting status (determined using previously published methods by Garriguet [47]), age, smoking, self-perceived health, blood pressure, diabetes, heart disease, cancer, osteoporosis, education, physical activity, income, BMI, immigrant status and weekend reference day. Note: Quintiles 2 and 4 are not presented for readability.

** The p-value represents the global p-value for a significant difference across quintiles of intake of total sugars (%E)

¥ significant difference compared to Q5 in post-hoc comparison with Bonferroni adjustment in Model 3 (p<0.05)

£ significant difference compared to Q3 in post-hoc comparison with Bonferroni adjustment in Model 3 (p<0.05)

BMI, body mass index; DFE, dietary folate equivalents; y, years

Supplemental Table S12. Comparison of intakes from macro- and micronutrients by quintiles of total sugars intake (% energy) in Males Aged 71y+*

**Males 70y+ (n=1105)**	**Q1**		**Q3**		**Q5**		
	**Mean**	**SE**	**Mean**	**SE**	**Mean**	**SE**	**p-value****
Energy (kcal)	**1770.6**	80.4	**1783.6**	45.7	**1727.2**	50.2	0.971
Carbohydrates (%)	**40.0**[¥£]	2.2	**48.8**[¥]	1.2	**59.3**	1.1	<.0001
Total Fibre (g/1000 kcal)	**9.2**	0.5	**10.2**	0.4	**11.0**	0.6	0.158
Total Sugars (%)	**8.0**[¥£]	0.3	**18.3**[¥]	0.1	**32.8**	0.8	<.0001
Natural Sugars (%)	**4.9**[¥£]	0.3	**10.2**[¥]	0.4	**17.7**	1.4	<.0001
Added Sugars (%)	**2.9**[¥£]	0.3	**8.0**[¥]	0.5	**14.9**	1.5	<.0001
Free Sugars (%)	**3.0**[¥£]	0.3	**9.1**[¥]	0.5	**17.9**	1.3	<.0001
Fat (%)	**34.2**[¥]	1.1	**32.1**[¥]	0.9	**25.5**	1.2	<.0001
Saturated Fat (%)	**10.7**	0.4	**10.5**	0.4	**9.0**	0.5	0.074
Monounsaturated Fat (%)	**13.1**[¥]	0.5	**12.1**[¥]	0.5	**9.1**	0.5	<.0001
Polyunsaturated Fat (%)	**7.4**[¥]	0.4	**6.7**[¥]	0.2	**5.0**	0.3	<.0001
Linoleic acid (%)	**6.2**[¥]	0.3	**5.6**[¥]	0.2	**4.2**	0.2	<.0001
Alpha-linonelic Acid (%)	**0.8**	0.0	**0.8**	0.0	**0.6**	0.1	0.258
Protein (%)	**17.5**[¥]	0.6	**15.8**[¥]	0.5	**13.6**	0.6	<.0001
Cholesterol (mg/1000 kcal)	**171.0**	23.8	**133.1**	10.5	**116.4**	8.0	0.011
Alcohol (%)	**8.3**[¥]	2.0	**3.3**	0.8	**1.6**	0.7	0.002
**micronutrients: per 1000 kcal**							
Vitamin A (ug RAE)	**321.9**	30.9	**465.6**	66.6	**388.6**	28.1	0.140
Thiamin (mg)	**0.9**	0.1	**0.9**	0.1	**0.8**	0.0	0.213
Riboflavin (mg)	**0.9**	0.0	**1.1**	0.1	**1.1**	0.0	0.008
Niacin (mg)	**22.5**[¥]	0.8	**20.0**[¥]	1.2	**16.6**	0.7	<.0001

Vitamin B-6 (mg)	**0.9**	0.0	**0.9**	0.0	**0.9**	0.0	0.731
Folate (ug DFE)	**238.9**	17.8	**246.1**	16.7	**223.1**	9.4	0.679
Vitamin B-12 (mg)	**2.1**	0.2	**2.7**	0.6	**1.8**	0.2	0.666
Vitamin C (mg)	**33.4**[¥£]	3.7	**52.8**	7.7	**90.5**	15.3	<.0001
Vitamin D (ug)	**2.9**	0.4	**3.3**	0.4	**3.1**	0.3	0.460
Calcium (mg)	**304.1**[¥£]	13.4	**422.3**	27.0	**457.6**	32.4	<.0001
Iron (mg)	**7.0**	0.4	**7.3**	0.2	**6.6**	0.3	0.256
Magnesium (mg)	**161.3**	5.3	**171.5**	5.1	**169.2**	6.6	0.346
Phosphorus (mg)	**657.7**	27.7	**705.7**	28.9	**657.6**	31.5	0.067
Potassium (mg)	**1362.8**[¥£]	51.9	**1564.9**	45.7	**1693.4**	68.9	<0.001
Zinc (mg)	**5.5**	0.4	**5.9**	0.5	**4.8**	0.3	0.031
Sodium (mg)	**1610.9**[¥]	71.8	**1536.7**	81.7	**1262.9**	68.7	<.0001

* Intake of macro- and micronutrients across quintiles of total sugars intake (as % energy) were compared using a general linear model (PROC SURVEYREG) including the following covariates: Model 3, mis-reporting status (determined using previously published methods by Garriguet [47]), age, smoking, self-perceived health, blood pressure, diabetes, heart disease, cancer, osteoporosis, education, physical activity, income, BMI, immigrant status and weekend reference day. Note: Quintiles 2 and 4 are not presented for readability.

** The p-value represents the global p-value for a significant difference across quintiles of intake of total sugars (%E)

¥ significant difference compared to Q5 in post-hoc comparison with Bonferroni adjustment in Model 3 ($p<0.05$)

£ significant difference compared to Q3 in post-hoc comparison with Bonferroni adjustment in Model 3 ($p<0.05$)

BMI, body mass index; DFE, dietary folate equivalents; y, years

Supplemental Table S13. Comparison of intakes from macro- and micronutrients by quintiles of total sugars intake (% energy) in Females Aged 71y+*

**Females 70y+ (n=1340)**	**Q1**		**Q3**		**Q5**		
	**Mean**	**SE**	**Mean**	**SE**	**Mean**	**SE**	**p-value****
Energy (kcal)	**1443.9**	77.3	**1401.1**	33.4	**1422.1**	35.9	0.914
Carbohydrates (%)	**41.8¥**	1.3	**48.2¥**	1.3	**59.9**	1.7	<.0001
Total Fibre (g/1000 kcal)	**9.8¥**	0.6	**10.6**	0.7	**12.6**	0.8	0.027
Total Sugars (%)	**8.4¥£**	0.3	**18.3¥**	0.2	**32.6**	2.9	<.0001
Natural Sugars (%)	**5.4¥£**	0.4	**11.0¥**	0.4	**18.7**	0.8	<.0001
Added Sugars (%)	**2.9¥£**	0.3	**7.2¥**	0.4	**13.8**	2.5	<.0001
Free Sugars (%)	**3.1¥£**	0.3	**8.8¥**	0.5	**16.9**	2.5	<.0001
Fat (%)	**36.4¥**	1.0	**32.6¥**	1.2	**25.9**	1.1	<.0001
Saturated Fat (%)	**12.1¥**	0.5	**10.8**	0.6	**9.0**	0.4	0.002
Monounsaturated Fat (%)	**13.6¥**	0.7	**11.7¥**	0.3	**9.2**	0.5	<.0001
Polyunsaturated Fat (%)	**7.4**	0.4	**7.2¥**	0.4	**5.4**	0.3	<0.001
Linoleic acid (%)	**6.3**	0.4	**6.1¥**	0.4	**4.5**	0.2	<0.001
Alpha-linonelic Acid (%)	**0.8**	0.0	**0.9¥**	0.1	**0.7**	0.1	0.013
Protein (%)	**18.9**	0.8	**16.4**	0.4	**13.5**	0.7	0.015
Cholesterol (mg/1000 kcal)	**186.2¥**	16.3	**144.2**	9.7	**110.8**	12.6	0.040
Alcohol (%)	**2.9**	0.9	**2.8**	0.7	**0.6**	0.3	0.039
**micronutrients: per 1000 kcal**							
Vitamin A (ug RAE)	**344.5**	43.0	**353.4**	36.3	**450.1**	38.7	0.382
Thiamin (mg)	**0.9**	0.0	**0.9**	0.0	**0.9**	0.1	0.655
Riboflavin (mg)	**1.0**	0.0	**1.0**	0.0	**1.1**	0.1	0.323
Niacin (mg)	**23.0**	0.8	**20.5**	0.6	**16.8**	0.8	0.014

Vitamin B-6 (mg)	**0.9**	0.1	**0.9**	0.0	**0.9**	0.0	0.531
Folate (ug DFE)	**226.3**	21.4	**238.5**	8.1	**230.0**	9.6	0.460
Vitamin B-12 (mg)	**2.3**	0.2	**2.0**	0.2	**1.8**	0.1	0.341
Vitamin C (mg)	**34.3**[¥£]	6.1	**57.4**[¥]	4.4	**93.0**	8.1	<.0001
Vitamin D (ug)	**2.5**	0.3	**2.7**	0.3	**3.2**	0.3	0.091
Calcium (mg)	**399.6**	18.8	**428.4**	20.2	**533.9**	45.8	0.004
Iron (mg)	**7.4**	0.4	**7.1**	0.2	**6.7**	0.3	0.617
Magnesium (mg)	**174.5**	5.6	**175.2**	6.4	**188.0**	10.8	0.682
Phosphorus (mg)	**737.8**	27.9	**687.1**	19.1	**718.4**	37.2	0.104
Potassium (mg)	**1507.5**[¥]	75.3	**1569.7**	46.4	**1799.3**	60.9	0.006
Zinc (mg)	**6.5**[¥]	0.4	**5.4**	0.2	**4.8**	0.3	0.021
Sodium (mg)	**1775.1**[¥]	84.5	**1558.0**	47.8	**1306.7**	71.4	0.003

* Intake of macro- and micronutrients across quintiles of total sugars intake (as % energy) were compared using a general linear model (PROC SURVEYREG) including the following covariates: Model 3, mis-reporting status (determined using previously published methods by Garriguet [47]), age, smoking, self-perceived health, blood pressure, diabetes, heart disease, cancer, osteoporosis, education, physical activity, income, BMI, immigrant status and weekend reference day. Note: Quintiles 2 and 4 are not presented for readability.

** The p-value represents the global p-value for a significant difference across quintiles of intake of total sugars (%E)

¥ significant difference compared to Q5 in post-hoc comparison with Bonferroni adjustment in Model 3 (p<0.05)

£ significant difference compared to Q3 in post-hoc comparison with Bonferroni adjustment in Model 3 (p<0.05)

BMI, body mass index; DFE, dietary folate equivalents; y, years
